# Genetic variant rs1058240 at the microRNA-binding site in the GATA3 gene may regulate its mRNA expression

**DOI:** 10.3892/br.2014.254

**Published:** 2014-03-14

**Authors:** FANG YANG, FENXIA CHEN, JUN GU, WENWEN ZHANG, JIAYAN LUO, XIAOXIANG GUAN

**Affiliations:** 1 Department of Medical Oncology, Jinling Hospital, Medical School of Nanjing University, Nanjing 210002, P.R. China; 2 Department of General Surgery, Jinling Hospital, Medical School of Nanjing University, Nanjing 210002, P.R. China

**Keywords:** breast cancer, *GATA3*, genetic polymorphisms, microRNA

## Abstract

The GATA binding protein 3 (GATA3) is a member of a family of 6 GATA dual zinc finger transcription factors (GATA1-6), which are required for the development and morphogenesis of the mammary gland. GATA3 is considered to play a dual role in oncogenesis and cancer development, whereas somatic *GATA3* mutations have been reported in breast cancer. Variants of the *GATA3* genetic 3′ untranslated region (3′UTR) microRNA (miRNA) binding sites have been associated with breast cancer risk. However, the roles of genetic variants in the *GATA3* gene 3′UTR and its post-transcriptional regulation have not been fully elucidated. We discovered that rs1058240 in the *GATA3* 3′UTR displayed potential miRNA binding sites and this variant was found to be significantly associated with *GATA3* mRNA expression (P=2.36E-07), suggesting that rs1058240 may be a putative variant mediating the post-transcriptional regulation of the *GATA3* target gene. Further studies investigating the regulatory mechanism of *GATA3* transcriptional activity are required to design novel strategies against breast cancer cell growth and differentiation.

## Introduction

The GATA binding protein 3 (GATA3) is a member of the GATA family of zinc finger transcription factors that bind to the consensus 5′-(A/T)GATA(A/G)-3′ motif (1). Human GATA3 exhibits 85% amino acid homology with human GATA1 in the DNA-binding domain, with no homology elsewhere in the protein, located on the 10p15 band of the human genome (2). GATA3, similar to other GATA family members, plays important roles in vertebrate embryonic organogenesis, including the mammary gland, sympathetic nervous system, parathyroid gland, kidney, inner ear, skin and T-cell lineages (3–9). Each GATA family member exhibits a distinctive pattern of expression in tissues and cell lines. The GATA3 protein is highly expressed in T-lymphoid cells and was suggested to be involved in the regulation of T-cell receptor α- and β-chain genes (2). GATA3 was identified in the luminal cells of mammary ducts and the body cells of terminal end buds, suggesting that GATA3 actively maintains luminal epithelial differentiation in the adult mammary gland, which suggests important implications in the pathogenesis of breast cancer (10). The majority of breast cancers arise from luminal epithelial cells; therefore, GATA3 appears to control a set of genes involved in the differentiation and proliferation of breast cancer cells. The expression of GATA3 is strongly associated with estrogen receptor (ER) expression in breast cancer (11) and there is accumulating evidence that GATA3 may be used as a clinical marker to determine response to hormonal therapy and refine the prognosis of breast cancer patients (12,13). The *GATA3* gene was recently identified as a potential tumor marker and putative tumor suppressor gene in breast cancer, whose expression may be associated with a more fvorable prognosis and prolonged disease-free survival in breast cancer patients (14). A meta-analysis reported that *GATA3* was one of the most significant genes exhibiting low expression in invasive carcinomas of the breast with poor clinical outcome, whereas low GATA3 expression was associated with a higher histological grade, positive nodes, larger tumor size, negative ER and progesterone receptor and HER2-neu overexpression (15).

To the best of our knowledge, microRNAs (miRNAs) may act as tumor suppressors and oncogenes by genetic variations in the 3′ untranslated region (3′UTR) binding sites, regulating the target-gene expression post-transcriptionally (16). Chou *et al* (17) demonstrated that GATA3 increased the level of expression of miRNA (miR)-29b, which in turn repressed a network of prometastatic microenvironmental components, including angiopoietin-like 4, lysyl oxidase, matrix metalloproteinase 9 and vascular endothelial growth factor A, through binding to specific sequence motifs in their 3′UTR. The realisation that the GATA3-miR-29b axis regulates the tumor microenvironment and inhibits metastasis may open up novel possibilities for therapeutic intervention in breast cancer. However, the role of genetic variations in the miRNA binding sites of *GATA3* has not been fully elucidated. Therefore, we tested our hypothesis that the *GATA3* 3′UTR variants may be associated with its mRNA expression by performing a bioinformatics analysis and genotype-phenotype association analysis based on the HapMap database.

## Materials and methods

### Bioinformatics and selection of polymorphisms

We identified the single-nucleotide polymorphisms (SNPs) in the *GATA3* gene and coding region by searching the National Center for Biotechnology Information online database (http://www.ncbi.nlm.nih.gov/SNP/). We limited the SNPs to those with a minor allele frequency (MAF) of >0.05 among different populations and used the SNP Function Prediction bioinformatics tool (http://snpinfo.niehs.nih.gov/snpinfo/snpfunc.htm) to predict the potential miRNA binding sites. We then calculated the genotype distributions of all the selected *GATA3* 3′UTR SNPs among different populations according to the database. In addition, the pairwise linkage disequilibrium (LD) values of all the SNPs in the same gene were calculated and the SNPs not in LD (r^2^<0.8) were selected. Subsequently, we plotted LD maps of those SNPs in *GATA3* gene with the LD TagSNP Selection online program (http://snpinfo.niehs.nih.gov/snpinfo/snptag.htm).

### Genotype and mRNA expression data of lymphoblastoid cell lines from the HapMap database

We used the data on *GATA3* genotypes and mRNA levels available online (http://app3.titan.uio.no/biotools/tool.php?app=snpexp) to analyse the genotype-phenotype association (18). The gene expression variation was analysed by using genome-wide expression arrays (47,294 transcripts) from Epstein-Barr virus-transformed lymphoblastoid cell lines from 270 HapMap individuals (128 females and 142 males) (19). The genotyping data from the HapMap phase II release 23 dataset consisted of 3.96 million SNP genotypes from 270 individuals belonging to 4 populations (20). The SNPexp v1.2 web tool (Norwegian PSC Research Center, Clinic for Specialized Surgery and Medicine, Rikshospitalet, Oslo University Hospital, Oslo, Norway) was used to analyse and visualize the correlation between HapMap genotypes and gene expression levels. The probe GI_4503928-S, representing the gene ‘*GATA3*’ was found in the file ‘illumina_Human_WG-6_array_content.csv’ and a correlation analysis was then performed between the SNP genotype and expression levels for the probe GI_4503928-S (additive model assumed).

### Statistical methods

We analysed the SNP genotype and phenotype correlation with the Chi-square test. The statistics test were two-sided and P<0.05 was considered to indicate a statistically significant difference.

## Results

### GATA3 3′UTR selected variants and putative miRNA binding sites

**Figure 1:**
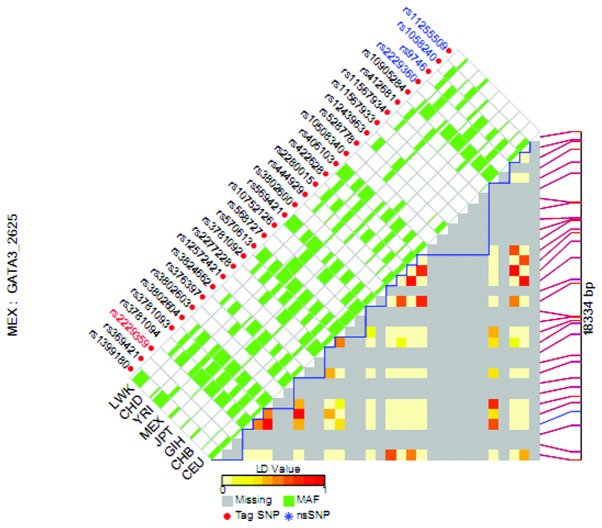
Linkage disequilibrium plot of the *GATA3* region using SNP Function Prediction (FuncPred). The color of each SNP spot reflects its D’ value, which changes from red to white as the D’ value decreases. SNP, single-nucleotide polymorphism; MAF, minor allele frequency.

In total, we identified 685 SNPs in the *GATA3* gene region and 73 in the coding region (http://www.ncbi.nlm.nih.gov/projects/SNP/snp_ref.cgi). Among these SNPs, 30 were located in the 3′UTR, of which 4 (rs2229360, rs58582188, rs9746 and rs1058240) exhibited a MAF of >0.05. The only SNP with putative miRNA binding sites revealed by SNP Function Prediction was rs1058240 (Table I). As presented in Table I, rs1058240 has three potential mRNA binding sites, including hsa-miR-1299, hsa-miR-182 and hsa-miR-95. We listed the genotype frequencies of 3 SNPs among different populations. rs58582188 was excluded, as it was not found in the database (Table II). We calculated the pairwise LD values of all the SNPs in the *GATA3* gene with a MAF of >0.05 and selected the SNPs not in LD (r^2^<0.8) to plot LD maps with the online SNP Function Prediction bioinformatics tool. The color of each SNP spot changing from red to white reflects the decrease in its D’ value (Fig. 1).

### GATA3 mRNA expression by genotype in lymphoblastoid cell lines

**Figure 2:**
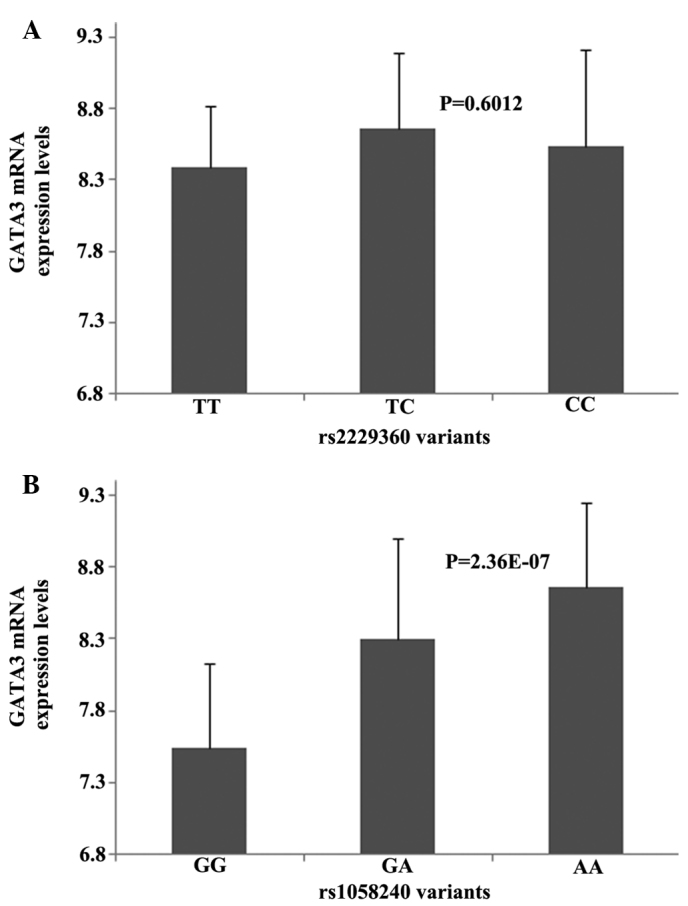
Genotype-phenotype association analysis of *GATA3* variants. (A) rs2229360 and (B) rs1058240 mRNA expression in Epstein-Barr virus-transformed lymphoblastoid cell lines from the HapMap database.

For the mRNA expression of the *GATA3* gene, we used the available HapMap-cDNA expression database for the correlation analysis of the *GATA3* genotype and mRNA expression level in Epstein-Barr virus-transformed lymphoblastoid cell lines from 270 HapMap individuals. For rs2229360, 268 cell lines with available values were collected. A total of 6 (2.2%) cell lines had the TT genotype, 42 (15.7%) had the TC genotype and 220 (82.1%) had the CC genotype. After excluding one cell line with unavailable values for rs1058240, 5 (1.9%) cell lines had the GG genotype, 62 (23.0%) had the GA genotype and 202 (75.1%) had the AA genotype. The *GATA3* mRNA expression levels of the cell lines with rs2229360 and rs1058240 are illustrated in Fig. 2. There was no significant difference in *GATA3* mRNA expression among the cell lines carrying rs2229360 variants (P=0.6012; Fig. 2A). The AA genotype of rs1058240 exhibited a significantly higher *GATA3* mRNA expression level compared to the GG and GA genotypes (P=2.36E-07; Fig. 2B).

## Discussion

The zinc finger transcription factor GATA3 was first identified in the early 1990s and is considered to be a marker of luminal breast cancers. GATA3 was confirmed to be necessary for the differentiation and maintenance of the luminal epithelium in the adult mammary gland (10). Usary *et al* (21) demonstrated that mutations in the second zinc finger may affect DNA binding, indicating its crucial role in the development and progression of breast cancer. *GATA3* was identified as one of the most significantly mutated genes in breast cancer by whole-exome sequencing and *GATA3* gene mutations were identified in 4 patients with luminal tumors, including 3 previously unknown frameshift mutations near the 3′-end of the coding sequence (22). *GATA3* mutations in breast cancer may be associated with loss of DNA binding, aberrant nuclear localization, decrease in transactivational activity and alterations in invasiveness, but not cell proliferation (23). It is well known that miRNAs may function as tumor suppressors and oncogenes through interactions with the 3′UTR of their mRNA targets and may control the target-gene expression post-transcriptionally (16). For example, GATA3 may promote differentiation, suppress metastasis and alter the tumor microenvironment in breast cancer by inducing miR-29b expression (17). It was also demonstrated that miR-30c was transcriptionally regulated by GATA3 in breast tumors (24). However, the roles of genetic variants in *GATA3* gene 3′UTR and its post-transcriptional regulation has not been fully elucidated. Our data demonstrated that rs1058240 located in the *GATA3* 3′UTR displays 3 putative miRNA binding sites by using bioinformatics analysis; this SNP is significantly associated with the mRNA expression level, suggesting it may be partly involved in *GATA3* post-transcriptional regulation. Our findings may enable a better understanding of the roles miRNA variants in *GATA3* 3′UTR play in its mRNA expression and open up novel possibilities for therapeutic intervention in breast cancer.

In conclusion, our results indicated the vital role of *GATA3* variants in 3′UTR in the post-transcriptional regulation of mRNA expression. However, the association of the regulation of *GATA3* transcription with variations in the 3′UTR requires further validation to facilitate the development of novel therapeutic strategies.

## Figures and Tables

**Table I:** Selected SNPs of 3′UTR and putative miRNA binding sites.

SNPs	Alleles	MAF	Putative miRNA binding sites
rs2229360	C/T	0.0845	NA
rs58582188	−/A/T	0.0964	NA
rs9746	A/G	0.1915	NA
rs1058240	A/G	0.1488	hsa-miR-1299, hsa-miR-182, hsa-miR-95

SNP, single-nucleotide polymorphism; 3′UTR, 3′untranslated region; MAF, minor allele frequency; NA, not available.

**Table II:** Frequency distributions of selected variables among different populations.

Genotypes	European	Asian	African
rs2229360			
CC	0.982	0.453	0.957
CT	0.018	0.453	0.043
TT	0.000	0.093	0.000
T alleles	0.009	0.320	0.022
rs9746			
AA	0.726	0.453	0.699
AG	0.257	0.453	0.265
GG	0.018	0.093	0.035
G alleles	0.0146	0.320	0.168
rs1058240			
AA	0.619	1.000	0.584
AG	0.336	0.000	0.363
GG	0.044	0.000	0.053
G alleles	0.212	0.000	0.235
